# Individual-specific associations between warble song notes and body movements in budgerigar courtship displays

**DOI:** 10.1242/bio.060497

**Published:** 2024-10-21

**Authors:** Nakul Wewhare, Anand Krishnan

**Affiliations:** ^1^ Indian Institute of Science Education and Research (IISER) Pune, Pashan Road, Pune 411008, India; ^2^ Evolutionary and Organismal Biology Unit, Jawaharlal Nehru Centre for Advanced Scientific Research, Jakkur, Bengaluru 560064, India; ^3^ Indian Institute of Science Education and Research (IISER) Bhopal, Bhauri 462066, India

**Keywords:** Budgerigars, Courtship, Multimodal coordination, Individual variability, Vocal sequences

## Abstract

Complex behavioral sequences such as courtship displays are often multimodal, and coordination between modalities is critically important. In learned and variable behavioural sequences such as songs, individual variability may also extend to multimodal coordination and the associations between modalities. However, individual variability in complex multimodal sequences and in coordination between distinct behaviours remains underexplored. Here, we report that budgerigars, which continuously learn and modify their complex warble songs, exhibit associations between body movements and song notes during courtship. Some associations are unique to individuals, and others are universal across individuals. Additionally, some individuals exhibit more unique associations than others. We also find that birds warbling in the absence of body movements emit all notes with broadly similar odds ratios. Our data suggest a hierarchy of associations, some individual-specific and others common to all individuals, between body movements and songs. We propose that these associations may be learnt and modified through social interactions, resulting in individual variability.

## Teaser

**Summary:** Budgerigars exhibit individual variability in associations between song notes and body movements during courtship. This suggests the possible influence of learning on multimodal courtship displays.

## INTRODUCTION

Multimodal communication is prevalent in the animal kingdom, serving various functions such as alarm signals and territorial defense displays (Ręk and Magrath, 2016). Courtship displays are frequently multimodal (Mitoyen et al., 2019). In avian species, courtship displays often combine acoustic and visual modalities, where songs are accompanied by elaborate body movements. This results in two distinct sequences: one of song notes and another of body movements (Dalziell et al., 2013; Rȩk, 2018; Rusiecki and Ręk, 2024; Scholes, 2008; Zsebok et al., 2021).

Animal vocalizations are often arranged into complex temporal sequences (Bhat et al., 2022; Engesser and Townsend, 2019; Kershenbaum et al., 2014, 2016), and their study sheds light into function and coordination in complex behaviours. Vocal sequences may be individual-, group- or species-specific, serving multiple functions from mate attraction to communicating group identity (Zsebok et al., 2021). Social learning introduces further lability, with sequences changing in species that learn throughout their lifetime (open-ended learners), a phenomenon very different from closed-ended learners, which may show some variation but do not add new elements in adulthood (Madabhushi et al., 2023a). Learning increases variability in sexually selected traits (Lachlan and Servedio, 2004), with consequences for evolution and speciation (Madabhushi et al., 2023b). When two complex behavioural sequences (for example, song and body movements) occur together, the signals can combine in various ways, leading to different perceptual consequences for the overall display. Broadly, they may either reinforce each other or combine to create a unique, new signal (Halfwerk et al., 2019; Partan and Marler, 1999; Ręk and Magrath, 2017; Rusiecki and Ręk, 2024).

In diverse avian courtship displays, studies have examined how components of song and body movements are associated with each other (Cooper and Goller, 2004; Dalziell et al., 2013; Fusani et al., 1997; Ligon et al., 2018; Miles and Fuxjager, 2018; Ota et al., 2015; Rusiecki and Ręk, 2024; Ullrich et al., 2016). Psittaciform birds or parrots are open-ended vocal learners, and continuously modify vocalizations to signal group identity (Farabaugh et al., 1994; Madabhushi et al., 2023a), in addition to courtship-related functions. Parrots are known to entrain their body movements to external beats (Hasegawa et al., 2011; Schachner et al., 2009; Seki, 2023); however, the higher-order associations of their flexible, complex vocal sequences with courtship displays and associated body movements remain unexplored. The presence of learning in both songs and body movements suggests that associations between the two may also be learned, and thus potentially variable across individuals or groups of individuals, but this variability has not been systematically investigated (Farabaugh et al., 1994; Mui et al., 2008).

**Fig. 1.**
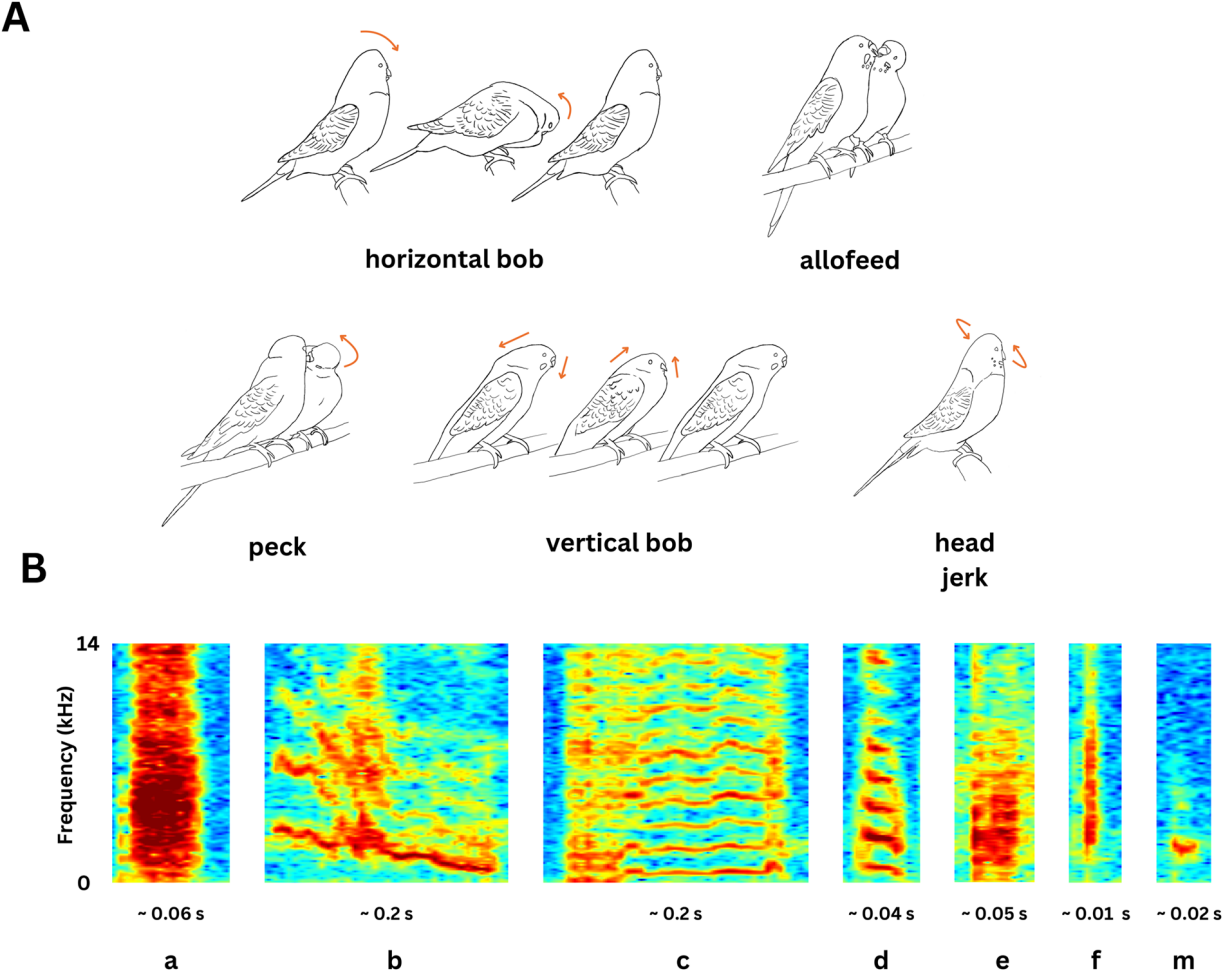
**(A) Graphical illustrations of different body movements of the male bird observed during display.** The direction of the arrows indicate the direction of future movement from that particular pose. (B) Spectrograms of the seven different note types in budgerigar warble song.

Here, we examined the association between courtship behaviours and song sequences in an open-ended learner, the budgerigar (*Melopsittacus undulatus*). Budgerigars possess a highly complex warble song, primarily studied in context of courtship (but also emitted in other social contexts), but which also serves as a socially learned signature of group identity (Farabaugh et al., 1994; Madabhushi et al., 2023a; Tu et al., 2011). Warble song may or may not be accompanied by body movements during courtship displays (Fig. 1A). Thus, it is an excellent system to understand how complex behavioural sequences are coordinated, and the social learning abilities of budgerigars also makes them an excellent system to address these questions. We aimed to understand whether body movements were associated with the emission of certain notes in the warble. Specifically, we focused on courtship-related body movements such as head bobs, jerks, pecks, affiliative behaviours including allofeeding and allopreening, and mounting behaviour.

We predicted that during courtship display, we should find significant associations between certain notes and body movements. Further, we predicted that birds warbling without body movements should not exhibit these predilections towards certain kinds of notes. Finally, given the complex patterns of learning and variability in budgerigar songs, as well as the literature on entrainment of movement to sounds, we predicted that associations between song and body movements should exhibit some degree of individual variability. Overlap or non-overlap of certain associations between different individuals would provide a preliminary indication that these associations can be learned socially and modified, similar to warble songs (Farabaugh et al., 1994; Madabhushi et al., 2023a).

## RESULTS

Our dataset of 100 courtship bouts across five males consisted of 19,934 annotated song notes and 2154 courtship-related behavioural events. The bout duration varied from 9–334 s (average 54.16 s). First, our basic count of co-occurrences demonstrated that notes and body movements were not obligatorily associated with each other and could occur independently (65% of notes occurred with a movement, as opposed to 35% occurring with body movements, whereas 91% of body movements occurred with a note, and only 9% without) (Fig. S1A). Although most body movements were accompanied by notes, this may have resulted from how we selected courtship bouts, where we initially searched for warble in the audio data, and then selected accompanying video. Nevertheless, this serves to demonstrate that warble song may occur independently of body movements, and that associations between them do not result from neural or mechanical constraints.

**Fig. 2.**
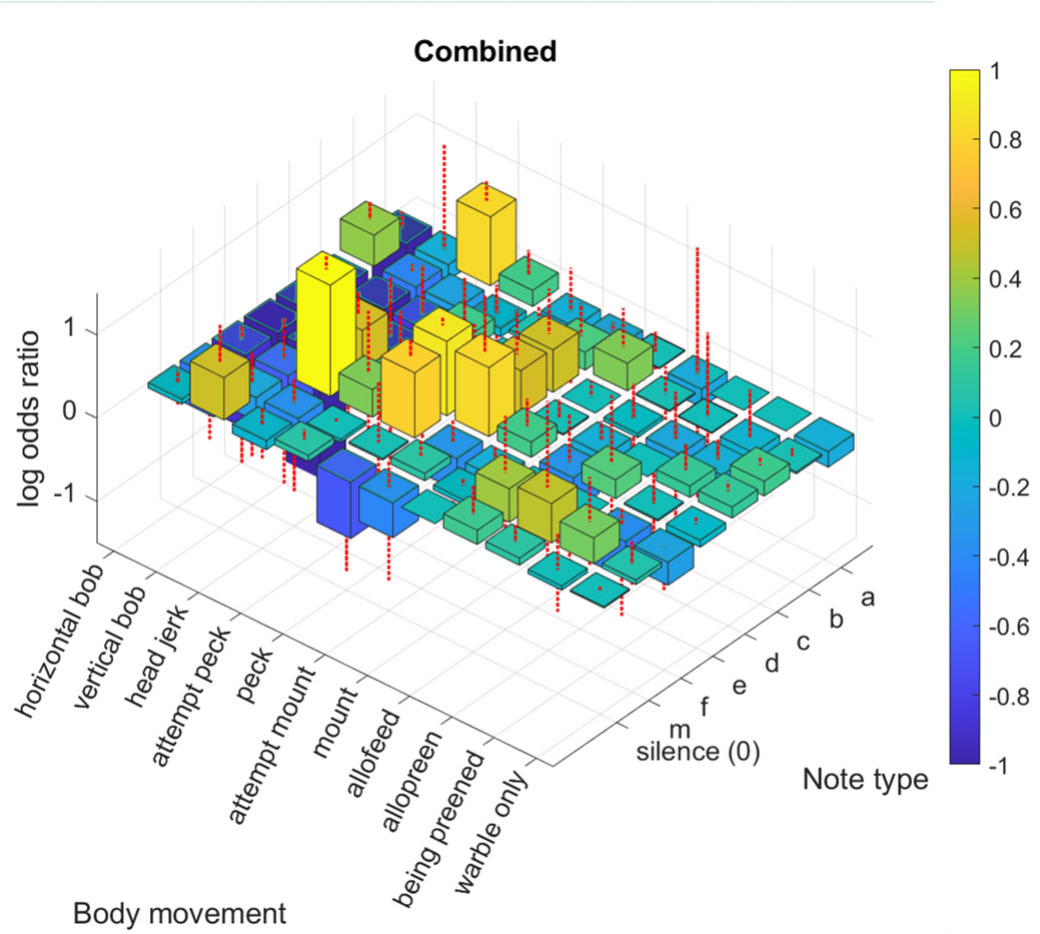
**Three-dimensional histograms showing the strengths of associations (log-odds ratio) between body movements and song notes for all birds combined.** Red dashed lines on the bars indicate standard deviations, and warmer colours indicate higher positive co-occurrence. The mean and standard deviation values used in the plot are provided in Table S2.

**Fig. 3.**
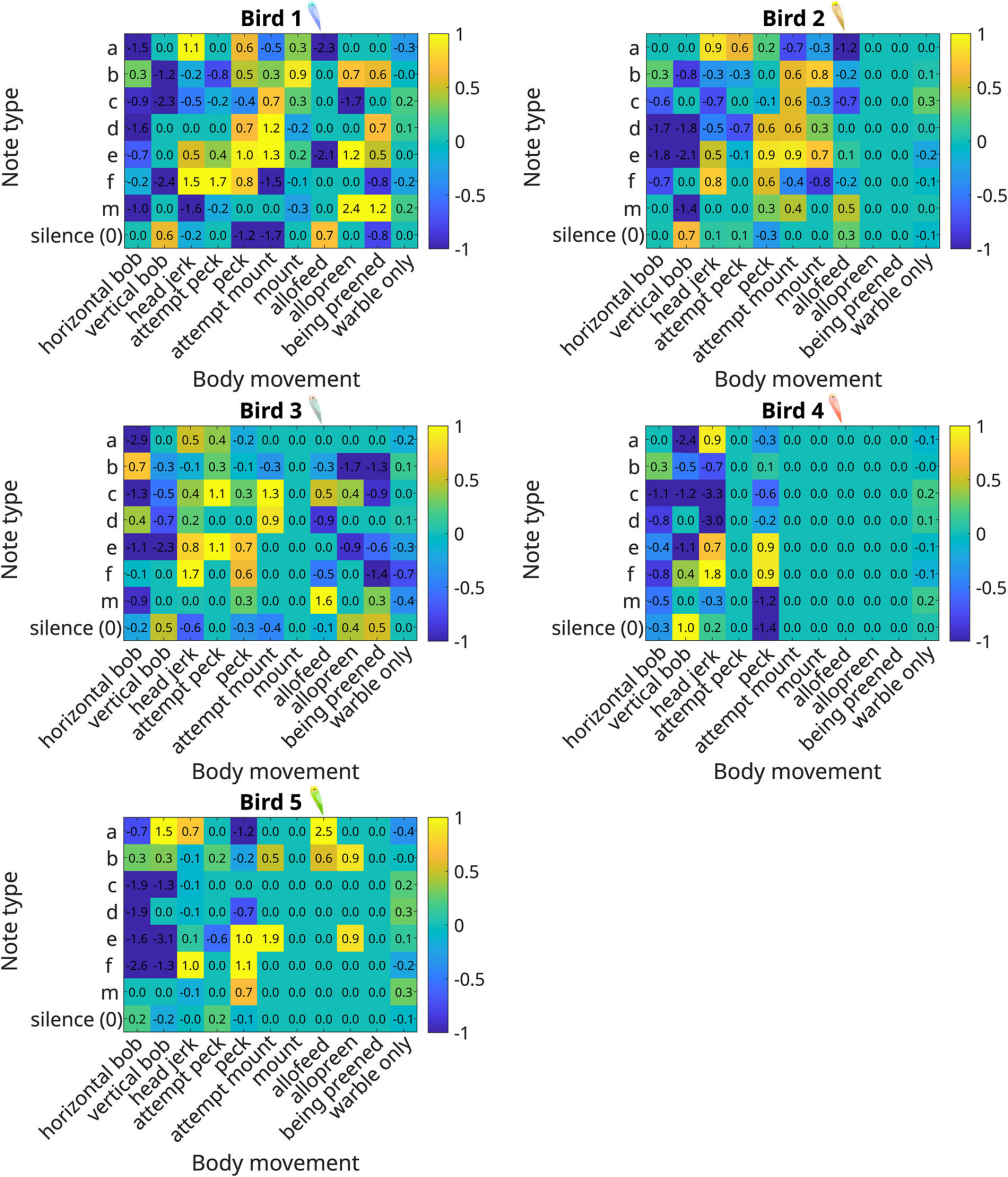
**Heat maps showing the strengths of associations (log-odds ratio) between body movements and song notes for each individual bird, with values provided in each square.** Note the variation in associations across individuals.

**Fig. 4.**
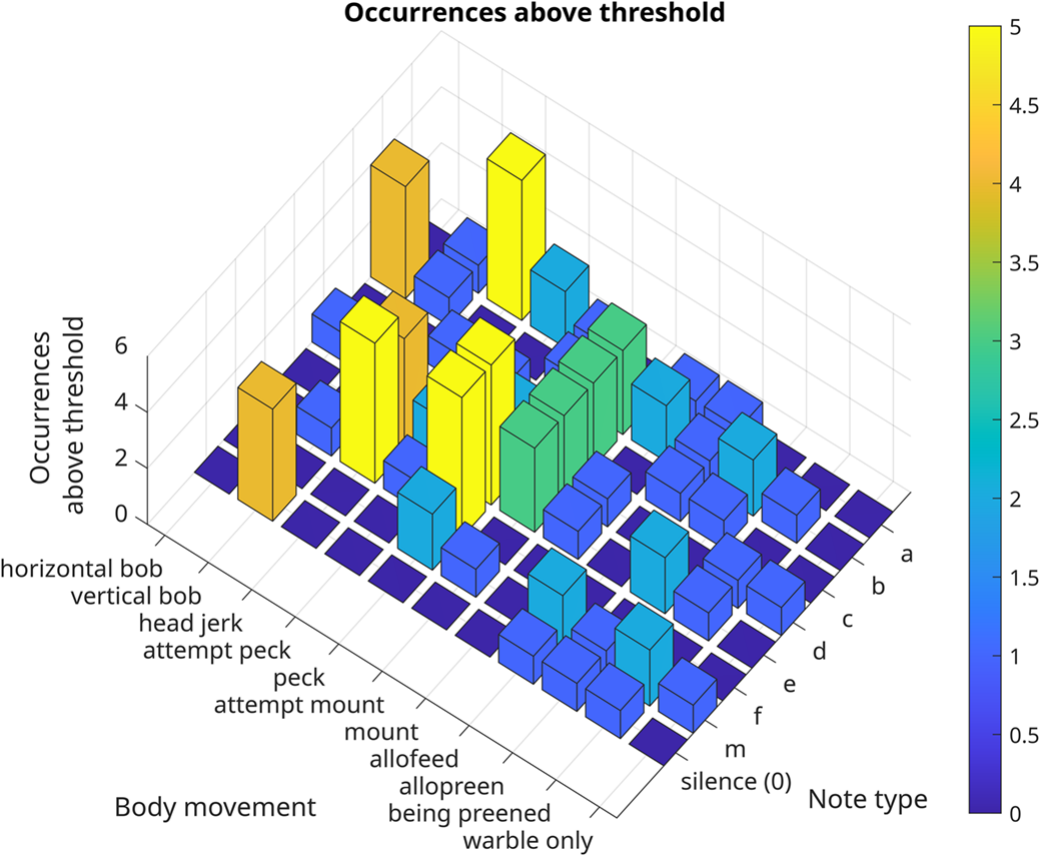
**Three-dimensional histogram showing the number of individuals (vertical axis, 0 to 5) exhibiting high log-odds ratios for a particular note-body movement association.** Only some associations are common to all five individuals.

**Fig. 5.**
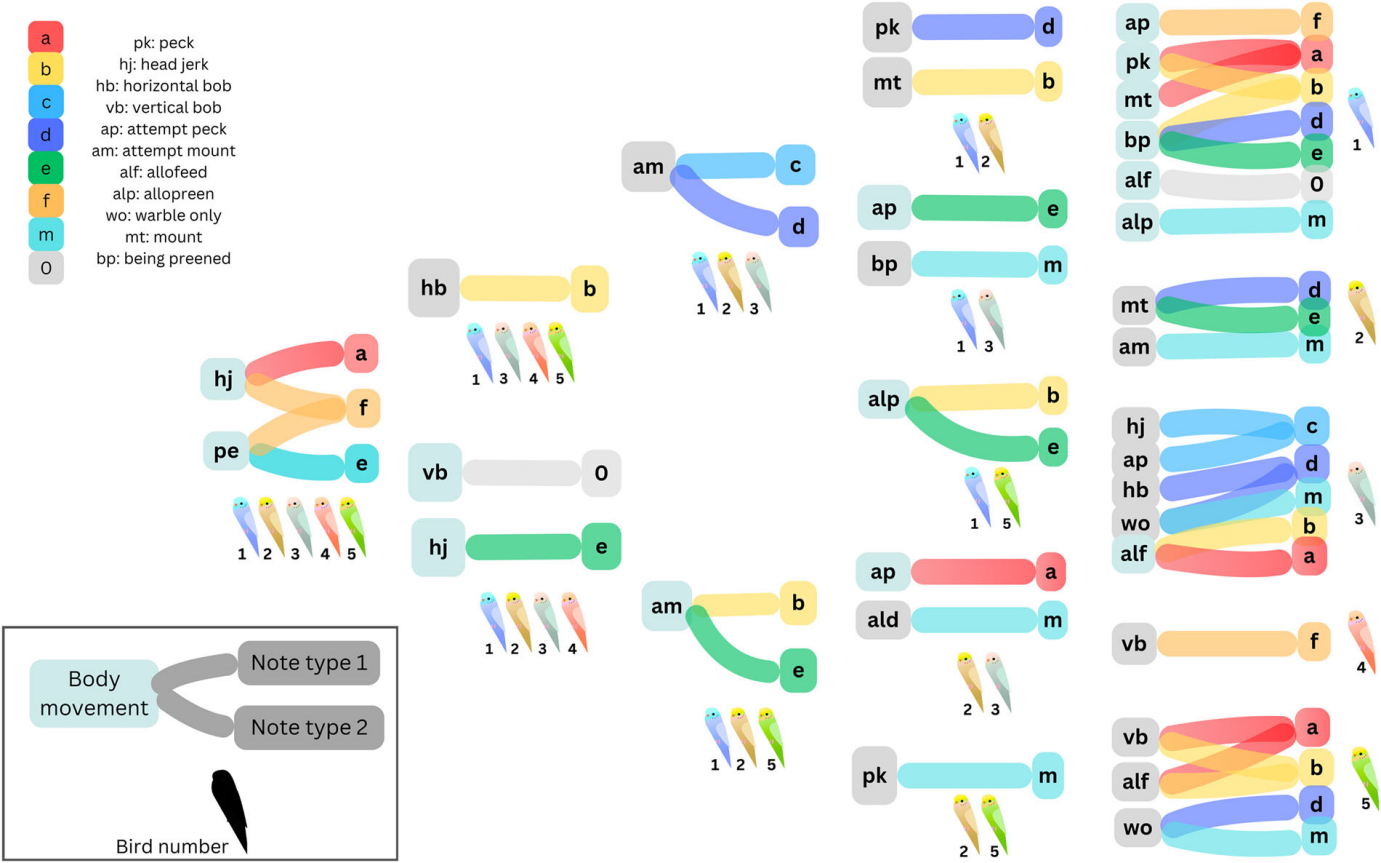
**Some associations between notes and body movements were shared across all individuals (left), whereas others were found in subsets of individuals or were unique to a single bird (right), as indicated by the illustrations under each.** The associations are represented as bars between different nodes, with nodes representing notes on the right side and body movements on the left side. Colours represent the note associated with the body movement. and the movements and notes are labelled according to the key in the top left. A key to interpreting this figure is also provided at the bottom left. Some body movements are associated with two or more notes, and thus have two or more bars emerging from them.

Our pointwise mutual information (PMI)-based analysis next sought to examine which notes were consistently associated with a body movement. All five males exhibited high co-occurrence for horizontal bobs with the b note, head jerks with the a and f notes, and for pecks with the e and f notes (Figs 2,3 and 4, mean and standard deviation in Table S2). Additionally, co-occurrence between other behaviours and song notes exhibited patterns specific to certain individual males or groups of males, resulting in a unique, nested pattern of relationships between body movements and vocal sequences for each bird (Figs. 3 and 5). Four males (birds 1, 2, 3, and 4) exhibited high co-occurrence of vertical bobs with silent periods and head jerks with e notes. Various other combinations of song notes co-occurring with body movements during courtship were specific to two or three males, and others to single individuals (Fig. 4 and 5). As a result, each individual male had a unique, hierarchical relationship between body movements and the notes comprising the warble song sequence, with some associations common to other males, and others unique to that male alone. Many combinations of body movements and notes also occurred with significantly negative log-odds ratios, meaning they were less likely to occur together than by chance. For example, in the combined data, the horizontal bob had a negative association with all notes other than ‘b’, and all birds exhibited a negative association between pecks and note type ‘0’.

Two other important patterns stood out here: when warbling without other courtship behaviours (‘warble only’), birds did not exhibit broadly increased associations with any note, largely emitting all notes with similar odds ratios (Figs 2 and 3). Second, the number of individual-specific associations varied between birds: some birds (1 and 5) exhibited multiple unique associations between notes and behaviours, whereas others (2 and 4) had very few, and tended to exhibit associations that were also found in other birds (Fig. 5).

## DISCUSSION

Individual variability in behavioural traits has received much study over the years. In particular, social context and social environment are known to influence behaviour, either by increasing or decreasing individual variability (Laskowski et al., 2022). Bird song is one of the more extensively studied examples of a learned behaviour, with functions in courtship and territorial defence (Marler and Slabbekoorn, 2004; Marler and Tamura, 1964). Avian vocal sequences exhibit considerable individual variability (Zsebok et al., 2021), which may serve important communicative functions (Engesser et al., 2016, 2019; Suzuki et al., 2018, 2019). The relationship between song and courtship display has received study in diverse birds such as cowbirds, zebra finches and lyrebirds (Cooper and Goller, 2004; Dalziell et al., 2013; Ullrich et al., 2016). In keeping with these studies, we demonstrate that budgerigar warble sequences and the corresponding body movement sequences are not independent of each other, as there are significant associations between specific notes and body movements. These associations are not obligatory, and birds frequently warble without body movements. This suggests that associations between song and movements do not arise from neural or biomechanical constraints. The specific associations and the total number of associations exhibited by an individual also vary. Based on these data, we propose a hierarchy: at one level, there are associations common to all individuals in a colony, followed by associations common to a subset, and finally, individual-specific associations. Thus, each individual has a unique combination of associations. Although the word choreography is often used to include steps that involve foot or head movements (for example, horizontal or vertical bobs in our data), we also found that budgerigars exhibit associations between song notes and other affiliative and copulatory behaviours associated with courtship, such as attempted mounting and allopreening. Our study presents evidence that associations between body movements and song notes may be individually variable (at least in open-ended learners such as budgerigars) along a hierarchy as detailed above, and that song produced during courtship is thus different from song produced without body movements.

The behaviour of warbling without any accompanying body movement, in contrast, was not associated with specific notes. Therefore, the sequences of warble and body movement potentially influence each other whenever a body movement occurs, whereas birds warbling without movement may emit any of the notes in their repertoire. We cannot determine which of the two sequences influences the other, as our data only demonstrates association with individual variability. However, the directionality of this influence is an interesting future topic of study. Given that the time scale of notes is much shorter than that of body movements, it is possible that movements act as states influencing which notes are emitted in the song. Note-movement associations could serve to attract the attention of females, especially in large flocks of conspecifics. Within these flocks, multiple males simultaneously display and vocalize. The associations between body movements and warbles in these scenarios may facilitate individual and group identification by female birds (Balsby et al., 2012; Smith-Vidaurre et al., 2023; Ullrich et al., 2016).

In budgerigars, it is plausible that associations between vocalizations and body movements could also be learned and modified over the course of a bird's lifetime in response to social factors. This hypothesis remains to be tested, however, and represents another interesting future line of research, especially because budgerigar songs are continually modified by social learning (Farabaugh et al., 1994; Madabhushi et al., 2023a). The pattern and hierarchy of associations observed are similar to patterns seen in other learned behaviours, where certain patterns are common and others vary individually (Janik and Slater, 2000). For example, birds that lived together in a colony might learn shared associations from each other. Finally, variations in note-movement associations among males could also result from female choice and female identity, where associations are driven by each female's preferences (Rusiecki and Ręk, 2024; Vallet and Kreutzer, 1995). In our experiment, two males shared a common female (birds 1 and 2), whereas all other pairs were unique. Birds 1 and 2 had the highest number of associations in common (16), with the second highest being 11 between birds 1 and 5. Although an interesting qualitative observation, this is very important to consider in further studies by pairing each male with different females. Further, many birds exhibited significant negative associations between certain notes and movements, which serves to further highlight the non-randomness of the warble and body movements, and also merits further study.

Warble song serves functions other than courtship, including communicating group identity through higher-order patterns of note repetition embedded in lengthy sequences (Madabhushi et al., 2023a). The portion of the warble song without any body movements is not particularly associated with any note type. Therefore, it could be used in contexts other than courtship, to convey other important signals. We propose that social learning modifies associations between song and behaviour, and this may result in signatures of both individual and colony identity. The complexity of these signatures may depend on social structure within a group. Thus, songs may vary at individual scales, colony scales and population scales, and this variation may include note structures (Dahlin et al., 2014; Farabaugh et al., 1994), repetition patterns (Madabhushi et al., 2023a) and their associations with distinct body movements (this study). Future studies will seek to disentangle these effects and the effect of learning on courtship display (Soma et al., 2019). More broadly, however, we highlight how the multifunctional budgerigar warble is a tractable system to study individual variability in complex behaviours, and its relationship to social structure.

## MATERIALS AND METHODS

### Study animals

All experimental procedures were performed on the Indian Institute of Science Education and Research (IISER) Bhopal campus, and were approved by the Institutional Animal Ethics Committee (protocol no: IISERB/2022/001) in accordance with the guidelines laid out by the Committee for the Purpose of Control and Supervision of Experiments on Animals (CPCSEA, New Delhi). We studied five male budgerigars (*Melopsittacus undulatus*) (henceforth referred to as birds 1, 2, 3, 4, and 5), who occupied a colony with three females and one bird whose sex could not be conclusively determined by cere colour. Birds were given *ad libitum* water and commercially available bird seed and maintained in a room at 25°C, as per a previously published study (Madabhushi et al., 2023a).

### Recording procedure

We recorded songs and courtship displays from male birds from 11-23 July 2022. Each male bird was placed in a cage within a soundproof acoustic enclosure of dimensions 75×75×75 cm (Newtech Engineering Systems, Bengaluru, India), with the bird to whom it exhibited the most affiliative behaviour and singing. To identify these pairs, we performed daily behavioural observation of the colony for a week prior to recording in the colony cage. During this time, the birds had no movement restrictions and could interact freely with other birds. After this pairing was identified, each male bird was paired with the same bird in all subsequent recordings. Three males were paired with unique birds, whereas one female was paired with the other two birds (males 1 and 2), as both males sang the most to the same female. Birds remained in the enclosure for 24 h to habituate them to the experimental paradigm. The cage, with dimensions of 55×35×45 cm, had three equidistant perches and one food and water cup. The camera and audio recorder were placed outside the cage.

Birds were video-recorded the next day from 8 AM to 6 PM using either GoPro Hero 5 or Hero 7 cameras (GoPro Inc., San Mateo, USA) at 60FPS/720p (Hero 5) or 100FPS/960p (Hero 7), to ensure a wide angle view no matter which camera was being used. We additionally recorded audio using Audiomoth recorders (Hill et al., 2019) at a sampling rate of 44.1 kHz. Each pair was recorded twice with an interval of one week between the recordings. During the period of our study, we did not detect warble songs or displays from females.

### Analysis

In order to understand the relationship between body movements (a complete list of movements along with their descriptions can be found in Table S1) and warble song notes during courtship displays, we first defined a courtship bout as beginning with either a note or a display step, and ending when neither had occurred for 2 s. The latter was based on how long a bird typically took to resume a display after changing perch. As a conservative threshold, we used double the threshold for ending a warble (1 s, from a previous study) (Madabhushi et al., 2023a) to identify the end of courtship bouts. We first went through the audio recordings and selected a random warble, then selected the accompanying videos and identified the start and end of the bout according to the aforementioned criteria. By assuming the start of the courtship bout to be either body movement or warble notes, we found warbles that were both accompanied and unaccompanied by body movements. This method of selecting courtship bouts provided information about associations between notes and body movements, if present.

We selected 10 such courtship bouts from each pair per day, one from each hour between 8:00 h and 18:00 h, giving us 100 courtship bouts in total from five males. Simultaneously, we classified warble elements into different note types, following the classification key employed by several previous studies (Farabaugh et al., 1992; Madabhushi et al., 2023a; Tobin et al., 2019; Tu et al., 2011) (Fig. 1B). These fell into the following categories: ‘a’, alarm call-like elements; ‘b’, contact call-like elements (frequency modulated sounds); ‘c’, long harmonic calls (harmonic sounds with duration greater than 100 ms); ‘d’, short harmonic calls (harmonic sounds with duration less than 100 ms); ‘e’, ‘noisy’ calls that are broadband and non-harmonic; ‘f’, clicks (extremely short broadband calls), and ‘m’, or ‘soft calls’. To incorporate the effects of silence into our analyses, we defined silent periods in the warble as instances where the inter-note interval exceeded 0.5 s, again following rationales established in published literature (Bhat et al., 2022; Madabhushi et al., 2023a,b). We labelled the silent periods in the warbles as ‘0’ in our note sequences. Audio annotations for the current study were done alongside the annotations for a previous study (Madabhushi et al., 2023a), and also verified simultaneously for interobserver agreement. In that study, three observers independently annotated 10 warbles and achieved an agreement rating of 80.16% using the normalized Levenshtein distance to calculate the agreement percentage, and the same value applies to this dataset as well.

We aligned the audio and video by matching spectrograms of sound from both sources in Raven Pro 1.8 (Cornell Laboratory of Ornithology, Ithaca, NY, USA) to give us the start of the bout in both channels. To obtain the start and end time of each note in the aligned recorder audio, boxes were plotted manually around each note using the spectrogram view in Raven Pro (default settings of window size 512 with 50 per cent overlap). For video data, we scored all courtship-related behaviours and their start and end times frame-by-frame using BORIS v8.6.5 (Friard and Gamba, 2016). A detailed description of each behaviour is given in Table S1, and some of the body movements are illustrated in Fig. 1A (also see Movie). To assess the objectivity of our body movement annotations, two observers independently annotated 10 randomly chosen courtship bouts in BORIS, two from each male (Bryington et al., 2002). We calculated Cohen's kappa using BORIS's inbuilt function, applying a time unit of 5 milliseconds (ms) (the same unit used in further analyses) for each bout separately, and obtained an average of 0.69, a maximum of 0.84, and a minimum of 0.61. These results indicated high agreement among annotations from multiple observers, supporting the reliability and objectivity of our body movement classification. Certain body movements, such as dilation of the iris and raising head feathers, were not included in our analysis because we could not reliably assign start and stop times based on our camera setup.

In addition, we noted periods of ‘warble only’, defined as no display element occurring for at least 1 s, but with warble song notes being emitted. After labelling our audio and video data, we obtained two separate time series of notes and behaviours in a courtship bout. First, we calculated the simple counts of co-occurrence of events in the two time series data, i.e. the number of times combinations of notes and body movements occurred together, as opposed to separately (Fig. S1A). We also calculated co-occurrence of notes or body movements with silent gaps in the other sequence (i.e. longer than 0.5 s in the warble, or one second in the movement sequence, as defined in previous paragraphs). Gaps shorter than this were not included in our analysis as we did not consider them biologically meaningful. We then obtained percentages for each note occurring with and without body movements, as well as percentages for movements occurring with and without notes. We discretized these into segments of 5 ms, shorter than the shortest note type (7 ms), thus ensuring that multiple events did not occur in a single segment. Next, we used the pointwise mutual information (PMI) framework for our measure of association between body movements and warble notes. PMI compares the probability of two events occurring together to the probability expected if the events were independent. Mathematically, PMI quantifies the discrepancy between the probability of two events coinciding, given their joint distribution and their individual distributions, and assuming independence (Bosshard et al., 2022; Church and Hanks, 1990). We quantified this discrepancy by calculating the odds ratio, which is obtained by dividing the observed number of co-occurrences by the expected number of co-occurrences, again assuming independence between the two events. This framework of PMI is commonly used in computational linguistics to quantify word associations.

We calculated the odds ratios of co-occurrence of each combination of note and body movement, for our time-discretized data. The expected value, assuming both time series are independent of each other, was calculated as:

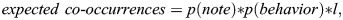
where *p(note)* and *p(behavior)* represent the probability of a given note and a given behaviour in a bout sequence, respectively, and *l* is the length of the bout sequence.

Next, we calculated the natural logarithm of the odds ratio for all possible combinations of notes and behaviours. Therefore, an odds ratio of 1 resulted in a log-odds of 0, implying that the note and the behaviour occurred together as often as expected by chance. A log-odds>0 implied a greater co-occurrence than expected by chance, and vice versa for values <0. To avoid the possibility of rare behaviours exhibiting disproportionately high odds ratios, we calculated the significance of each of our observed log-odds using a permutation test as described below, and only considered significant co-occurrences for further analysis.

First, we randomized our audio and video time series 10,000 times and then calculated log-odds for all combinations of notes and behaviours in each randomized iteration. This gave us a distribution of log-odds for each combination. We calculated two-tailed *P*-values by counting the number of randomized log-odds with absolute values greater than the absolute observed log-odds for each combination. We used two-tailed *P*-values to account for both significantly high and low log-odds. To correct for multiple testing on the same data, we controlled the false discovery rate using the Benjamini–Hochberg procedure. Results reported as significant assumed a false discovery rate of 0.05. Moving forward, we only considered significant log-odds for further analysis, whereas all non-significant log-odds were set to zero. To set more reliable criteria for defining high log -odds, after selecting significant log-odds ratios, we applied a threshold to identify values above this threshold. We compiled all the log-odds from all individuals, sorted them by their values, and identified the top 20th percentile of the data, which yielded a log-odds threshold of 0.285 (Fig. S1B). Therefore, any log-odds value above this threshold was considered high in our subsequent analysis. All values above this boundary were treated as strong or significant associations, to simplify interpretation.

## Supplementary Material

10.1242/biolopen.060497_sup1 Supplementary information

Table S3.
