# Supplementary material for: Individual-specific associations between warble song notes and body movements in budgerigar courtship displays
Source: Biol Open. 2024 Oct 21;13(10):bio060497. doi: 10.1242/bio.060497 (PMC11552614; doi:10.1242/bio.060497)
Supplement: Supplementary information [file biolopen-13-060497-s1.pdf]

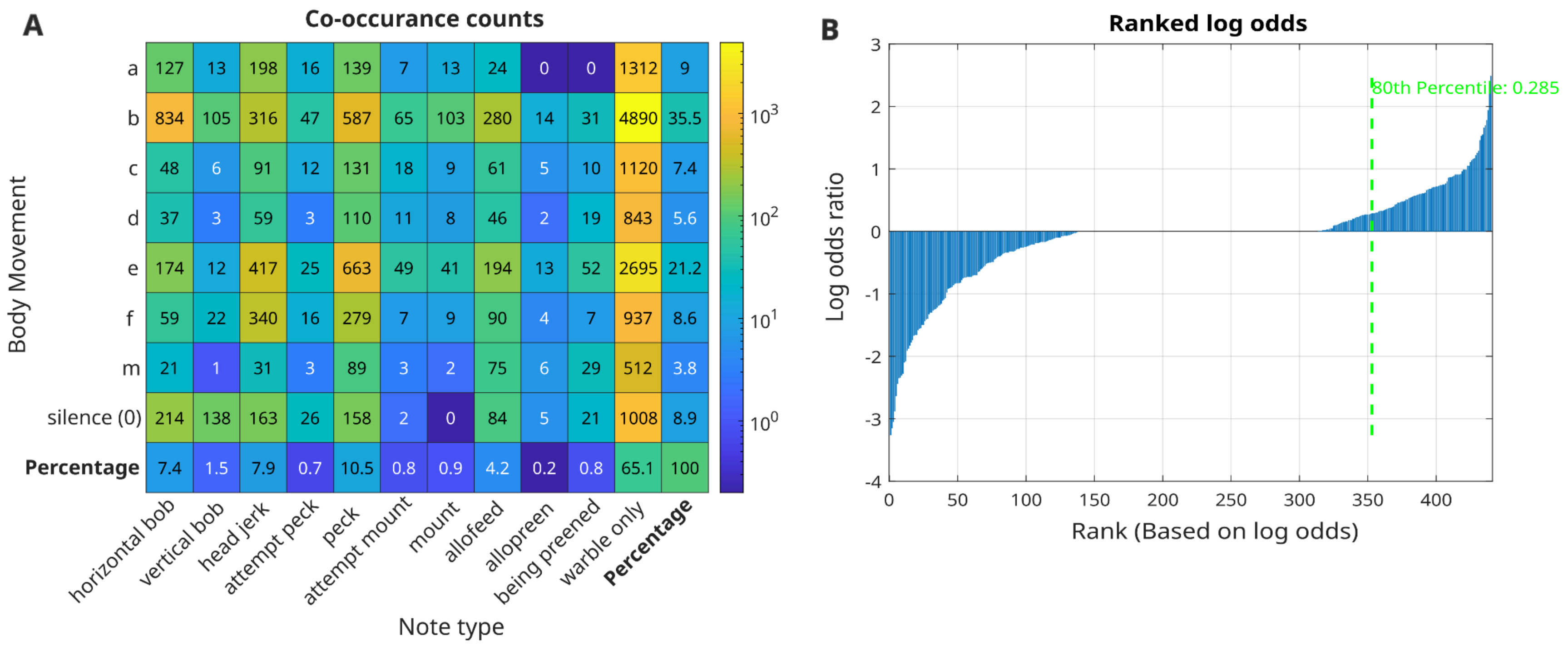

Fig. S1. A. Raw co-occurrence counts for all combinations of notes and body movements, along with the percentage of the overall repertoire comprised by each, in the last row and column of the matrix. **B.** Ranked distribution of log-odds, with a cut-off of at 80th percentile, which is value of 0.285 indicating a significantly high log-odds.

Table S1. Categories of behaviors analysed from video data, with information on how they were identified and scored. Note: if a display behavior repeats itself again in a series, for example four continuous horizontal bobs, all of them are taken as a horizontal bob event, with start taken as start of first bob and end taken as end of last bob.

**Behavior**	**Description**	**Start**	**End**
horizontal bob	the male lowers its head below or in line with the level of its torso in front of its body axis, and subsequently raises it back	closing of eye lids before lowering the head	opening of eye lids after returning to resting state
vertical bob	the male lowers its head along the body axis by retracting its neck, extends the head above its resting position, and then returns it to its original resting state	closing of eye lids before lowering the head	opening of eye lids after returning to resting state
head jerk	the male rapidly jerks its head, making a 180-degree rotation, all while puffing its head feathers. Alternatively, it can quickly jerk its head from one side to the other.	start of the first movement	end of the last movement
attempt peck	the male attempts to peck the female bird, but does not make physical contact, for example because the female moves away	start of the motion of male's head towards female to peck	return of head to resting position
peck	the male bird moves its head towards the female, briefly making contact with her using its beak, before returning its head to its resting state.	start of the motion of male's head towards female to peck	return of head to resting position
attempt mount	the male attempted to mount the female bird, but the female moved away, preventing successful mounting	male raises its leg to mount female	both legs return to the ground
mount	male bird mounts female bird	male raises its leg to mount female	both legs return to the ground
allofeed	the male regurgitates food into the mouth of the female	movement of male's head towards female to allofeed	return of head to resting position
allopreen	the male bird preens the female bird	movement of male's head towards female to preen	return of head to resting position

Biology Open • Supplementary information

being preened	The male bird is being preened by the female bird, this is included as male behavior because the male participates in the behavior and vocalizes.	movement of female's head towards male to preen	return of head to resting position
warble only	The male bird continues to warble but remains stationary and does not exhibit any other behavior.	If no behavior is observed for 1 second, but the warble continues uninterrupted, the starting time will be the time of the first note that appears after the last observed behavior.	If there is no new note in the warble song for 2 seconds, or if the start of some other behavior is observed,

Biology Open • Supplementary information

Table S2. The mean and standard deviation of the log odds ratio for combined data from all birds. The mean is on the left and the standard deviation on the right.

	horizontal bob	vertical bob	head jerk	attempt peck	peck	attempt mount	mount	allofeed	allopreen	being preened	warble only
**a**	-1.02, 1.21	-0.18, 1.43	0.83, 0.22	0.19, 0.28	-0.18, 0.69	-0.25, 0.35	0.01, 0.23	-0.21, 1.79	0.00, 0.00	0.00, 0.00	-0.19, 0.15
**b**	0.37, 0.19	-0.49, 0.56	-0.30, 0.25	-0.10, 0.42	0.04, 0.26	0.21, 0.38	0.33, 0.46	0.02, 0.32	-0.01, 1.01	-0.14, 0.70	0.02, 0.08
**c**	-1.17, 0.47	-1.08, 0.89	-0.84, 1.42	0.18, 0.49	-0.17, 0.34	0.51, 0.53	0.00, 0.18	-0.05, 0.45	-0.26, 0.81	-0.18, 0.40	0.18, 0.10
**d**	-1.13, 0.95	-0.50, 0.78	-0.67, 1.36	-0.14, 0.32	0.06, 0.57	0.53, 0.52	0.02, 0.21	-0.17, 0.38	0.00, 0.00	0.14, 0.30	0.13, 0.10
**e**	-1.10, 0.60	-1.72, 1.21	0.53, 0.27	0.17, 0.63	0.89, 0.10	0.83, 0.84	0.18, 0.32	-0.40, 0.95	0.23, 0.81	-0.02, 0.37	-0.08, 0.16
**f**	-0.89, 1.04	-0.65, 1.14	1.34, 0.45	0.33, 0.74	0.78, 0.20	-0.38, 0.65	-0.18, 0.36	-0.13, 0.22	0.00, 0.00	-0.45, 0.65	-0.27, 0.27
**m**	-0.48, 0.47	-0.28, 0.63	-0.41, 0.67	-0.04, 0.08	0.02, 0.71	0.09, 0.20	-0.06, 0.13	0.40, 0.67	0.47, 1.06	0.31, 0.53	0.05, 0.26
**silence (0)**	-0.06, 0.21	0.51, 0.43	-0.11, 0.31	0.07, 0.10	-0.68, 0.60	-0.42, 0.72	0.00, 0.00	0.17, 0.33	0.09, 0.19	-0.05, 0.47	-0.02, 0.05

Table S3.

Available for download at
https://journals.biologists.com/bio/article-lookup/doi/10.1242/bio.060497#supplementary-data

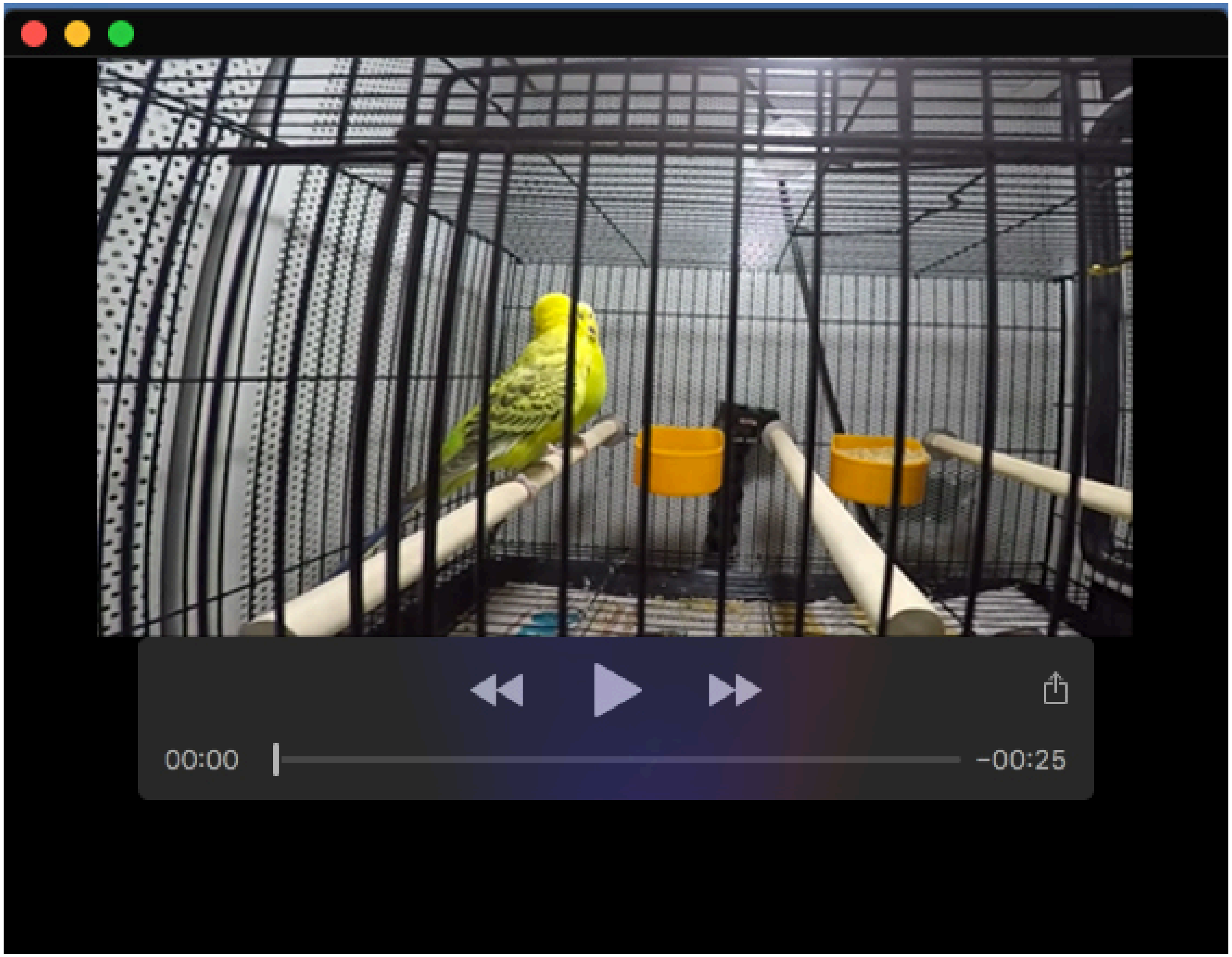

Movie 1.
